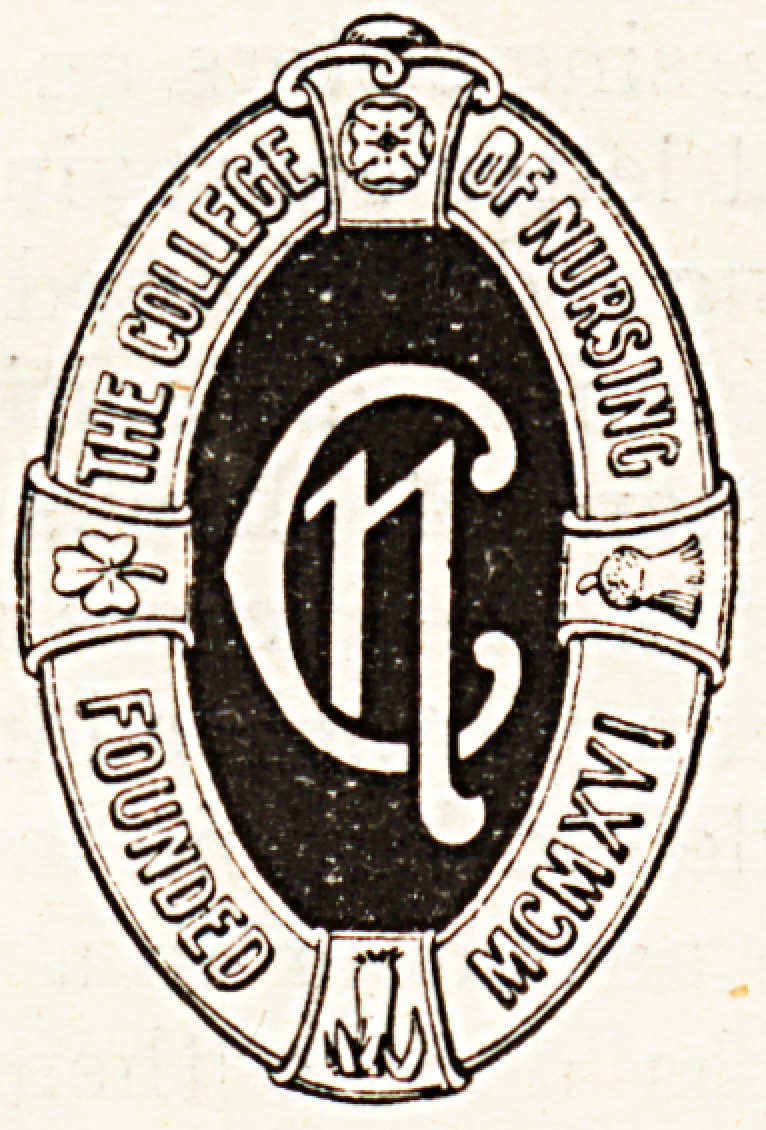# Round the Hospitals

**Published:** 1920-02-21

**Authors:** 


### ROUND THE HOSPITALS.

Ireland leads the way in the prompt appointment of its first General Nursing Council under the Registration Act. The Chief Secretary announces the following appointments to this Council: Dr. Coey Biggar, Chairman Irish Public Health Council; Countess of Kenmare, Vice-President of Q.V.J.N.I.; Colonel Sir Arthur G. Chance, Mater Misericordium Hospital, Dublin, and President of the Irish Board, College of Nursing; Colonel W. Taylor, C.B., Meath Hospital, Dublin, ex-President of the Royal College of Surgeons, Ireland; R. J. Johnston, F.R.C.S., Victoria Hospital, Belfast,

**ROUND THE HOSPITALS—*(continued)*.**

Chairman of Irish Medical Committee; Dr. Sullivan, Cork. The nurse members of the Council are the following: Miss Matheson, Secretary of the Irish Board, College of Nursing; Miss Reeves, Matron, Dr. Stevens's Hospital; Miss Mitchie, Superintendent, Q.V.J.N. Home, Dublin; Miss Huxley, Matron, Elpis Private Hospital, Dublin; Miss O'Flynn, Lady Superintendent, Children's Hospital, Dublin; Miss Bostock, Matron, Royal Victoria Hospital, Belfast, Irish Board, College of Nursing; Miss Walsh, Nurses' Training School, Waterford; Mrs. Blunden, Superintendent, Mosaphir Private Nursing Home, Cork; Miss Curtin, Lady Superintendent, Mater Infirmorum Nurses' Training School, Belfast, Irish Board, College of Nursing.

Lady Doreen Long has presented an Army Hut, formerly used at the Avon View Red Cross Hospital, to the Trowbridge Cottage Hospital, for the use of the matron and nurses. The gift of a strip of land, adjoining the hospital, by Mr. John Kemp, has enabled the hut to be placed in its own garden in quiet surroundings. It has been very prettily fitted up and furnished and affords a sitting-room, two bedrooms, and bathroom. The former accommodation for the staff is to be utilised for private patients. The hospital has gone ahead since the days when there was no surgery, no instruments, and no operation room. The authorities have done wisely in settling the nursing staff into suitable quarters before further extending the accommodation.

The news that Lady Muriel Paget has offered the Government of Latria to establish a children's hospital at Dvinsk, and that the offer has been gratefully accepted, will cause great satisfaction to many of our readers, who cannot rid their minds of the myriad starving little ones dying without succour. At Dvinsk the population is suffering greatly from scarcity, and fevers abound. The new hospital is to contain 100 beds for in-patients, and an out-patient department equipped to deal with 500 children.

There comes again an insistent call for help from the typhus-ridden countries. This time it is Southern Russia, where the population of the evacuated districts are in an appalling condition. Drugs, clothing and comforts of every description are required at once to succour the thousands attacked by typhus. The British Committee of the Russian Red Cross (35 Albemarle Street, W. 1.) appeal urgently for gifts in kind and money. All goods sent are distributed on the spot by the representatives of the British Committee, who are sending urgent telegrams for more help.

Best congratulations to the South African Trained Nurses' Association, which now numbers its first thousand members and a few to spare. This is a very fine record of progress in the course of five years, and testifies to the value the South African nurses attach to union. The difficulties of organising this Association, which started with a membership of less than a hundred, have been enormously increased by the war conditions, under which about a third of the present members were on active service. The number of resignations may, we are informed, be counted on one's fingers. The white population of South Africa totals only about a million and a-half, so that there cannot, it is estimated, be many nurses outside the ranks of the members. Supposing the proportion of trained nurses to the population to be similar in this country, the members of the College of Nursing ought not to number less than 29,000.

The *Daily Telegraph* Fund has now reached a total of 137,392 shillings. Among the welcome contributions we note one of 2,000 shillings from the National Sunday League. The contributors represent all classes and interests. A mere recapitulation of the names of a few among the donors suffices to show the hold which the nursing profession has on the public at large. Mr. George Robey, O.B.E.; Officers' Mess, Seaforth Highlanders; Staff of Messrs. Liebig's; Officers 2nd Batt. A.P.W.O. Yorkshire Regiment; Dominion Bank London; 3rd Batt. Yorkshire Light Infantry Officers; "In memory of our two boys in the K.R.R.C."; Officers 1st Batt. Lincolnshire Regiment; Sergeant's Mess Depot West Yorkshire Regiment; "In gratitude for the brave nurses' services to our village boys"; Farrow's Bank; Staff and girls County School, Folkestone; Sergeants of the 2nd R. W. Kent Regiment; J. Lyons and Co.; Beddington Working Men's Club; 1st Leicester Regiment, B. Company; Staff Collection at 7 Angel Court, E.C.; H.M.S. *Pegasus*; perhaps most significant to all—"A Blinded Soldier." The testimony of Mr. Alfred Humphreys, for nearly twenty years a member of the Holborn Board of Guardians, is particularly interesting. As one who knows the value of the services of "these angels of mercy" he urges the rich to come forward with their large contributions in aid of this laudible object. We reprint on another page the first list of the nurses' contributions, which total the splendid sum of 18,421 shillings. To quote the *Daily Telegraph*, "It is a result of which they might well be proud, seeing how small are their earnings."

A grand bazaar is announced to take place in the Great Hall of St. Bartholomew's Hospital on March 4, under the gracious patronage of Queen Alexandra, in aid of the funds. The occasion will be the first of its kind since the founding of the hospital in 1125. All articles on sale will be gifts, and a number of valuable pictures have been promised. Mr. George Robey has promised a performance, and the Earl of Granard's orchestra will play during the proceedings. The nurses' stall already promises well, and contributions from former Bart's nurses will be warmly welcomed.

**ROUND THE HOSPITALS—***(continued)*.

We are requested to call attention to the opening early in March of the valuable three months' electrical course at the Hospital for Epilepsy and Paralysis, Maida Vale, W. 2. Full particulars can be obtained from the Secretary of the Hospital.

---

Members of the College of Nursing can now have their own badge, of which we give an illustration. It is made in solid silver, with a royal blue enamel background encircled by a silver collar, and the (College) registered number is engraved on the back.

It can be obtained, from the beginning of March, for 4s. 6d., including postage, on application to the Secretary of the College of Nursing, 7 Henrietta Street, W. 1. Members must give their registration number in the College Register, which will be engraved on the back of the badge.

---

Ireland would appear to be waking up to the scandal of the nurse's conditions of service and her outlook. On Friday of last week Dr. Ninian Falkiner delivered an enlightened and informative address before the Statistical and Social Inquiry Society of Ireland, entitled "The Nurse and the State." The lecturer sketched the evolution of nursing in Ireland, pointing out that in 1872 there were no trained nurses in the country. But the burden of his mission was to endeavour by practical suggestions to deal with the inability of the impoverished middle classes to employ nurses now in their own homes owing to the inflated cost of living. Dr. Falkiner's solution of the problem is the extension of the functions of the district nurse, coupled with the betterment of her position, and in this connection he pointed to the support given to district nurses by the working classes in England, which is absent in Ireland. The creation of an advanced diploma in nursing science was recommended, so as to supply a staff of community nurses to administer the numerous activities of a public health department. On the part of the public, he said that it is a public duty to provide that the remuneration of the trained nurse is adequate, that her working hours are reasonable, that her holidays are sufficient for the recouping of her health, and that a suitable pension should be provided for her when her nursing skill becomes impaired. Ireland is waking up.

---

The quarterly meeting of the Association of Hospital Matrons was held, by kind permission of the Chairman and Committee, at the General Hospital, Birmingham, on February 7. Miss Lloyd Still, R.R.C., presided, and the large attendance of members from all parts of the country was satisfactory evidence of the vigour and interest which this new Association has called forth. The formation of Local Groups, each with its own delegate, is at present under consideration. Four such Groups are being formed experimentally, and if they prove a useful development in the work of the Association other centres will be established in due course. After the business meeting Miss Musson gave a most interesting account of her tour in Norway, illustrated by some beautiful slides, and subsequently all present were entertained to tea by the Midlands Group of Hospital Matrons. The annual meeting of the Association will be held in London about the middle of April.

---

Miss Hill, the matron of Adelaide Hospital, Dublin, is making a valiant effort to clear away the burden of debt that the war has caused. Prior to 1914 the Adelaide was in a flourishing condition, or, at all events, it paid its way, and often had a balance in the bank. But since that date soaring prices have doubled the cost of maintenance, and as the poor have all along been admitted free, the net result is a debt of £10,000. Miss Hill, in order to meet the deficit, issued an appeal for a hundred thousand half-crowns, and succeeded in enlisting the services of a small but zealous army of helpers. This task for a matron already very fully occupied is no light matter. In fact, it is a very heavy matter, calculated to intimidate the most stout-hearted. Happy to record, Miss Hill is succeeding. Already a quarter of the debt is cleared. The remaining three-quarters will also be cleared, and without delay, if the Protestant clergy of Ireland are willing to help. The Adelaide is a Protestant institution, and receives patients from every part of the island. Catholic hospitals are liberally supported by their own people. In many cases nuns are in charge, and they have but to ask in order to receive.

---

The Adelaide Hospital, Dublin, deserves support as an up-to-date institution. In spite of its depleted exchequer, it was the first hospital in Ireland to abolish a training fee for probationers, and although it bears a sectarian reputation, and is run by Protestants in the interests of the Protestant sick poor, no questions are asked as to creed when the sick ask for admission. Miss Hill is an Englishwoman, trained in the London Hospital, and proud of her training. She is possessed of many talents. She is now engaged in an up-hill fight, and she is winning. But her ultimate success will depend on the support of the Protestant community of the three southern provinces of Ireland. Ulster has its own institutions separate and distinct. The Church of Ireland, Methodist and Presbyterian communities of Munster, Leinster, and Connaught look to the Adelaide for asylum and treatment for their suffering sick poor. Now is the moment for a demonstration of practical sympathy. When the

**ROUND THE HOSPITALS**—*(continued)*.

debt is cleared there will be no further appeal. The Governors are formulating a scheme of self-support which will be satisfactory to all concerned, and which will put an end to the spectre of debt.

---

The conditions, sanitary and administrative, prevailing in some Irish Poor-law institutions have long been a matter of profound dissatisfaction, alike to the medical authorities, the nursing staff, and the inmates. There are many signs that the hour for reform has struck, and we welcome the forward movement for abolishing the "workhouse" element and remodelling the infirmaries on hospital lines. The Banbridge Guardians have recently voted the sum of £15,000 to remodel the fever hospital and convert the workhouse into a modern infirmary. Its present sanitary arrangements have been condemned by the Local Government Board inspector as "most primitive and unsatisfactory, a disgrace to any institution."

---

The opposition to Colonel Goodall's introduction of female nurses into the Cardiff Mental Hospital goes smouldering on. As medical superintendent of the hospital he attended a meeting of the Committee at the City Hall recently, and stated that he had not received a single complaint from any of the nurses of annoyance experienced by them in the course of their duties amongst the patients. A letter was read from the Trades and Labour Council reiterating former statements and declaring that the presence of female nurses in the wards during the war had an exciting and unhealthy effect on the patients. It was decided to ask the Trades and Labour Council to formulate their objection and present the Hospital Committee with definite data, the truth of which could be tested. Colonel Goodall complained that they were being kept in a ferment by these general allegations.

---

A delightful "at home" was held at the Wharncliffe Rooms, Great Central Hotel, on the 11th inst., by Miss Beardsmore Smith, R.R.C., Matron-in-Chief of Q.A.I.M.N.S., and other members of the Service, when many of those who gave their services in various departments of hospital work, under the Red Cross and the Order of St. John, were invited. The brilliant display of ribbons, badges, and orders, many bestowed by foreign potentates or republics, made the occasion unique. All the hostesses and most of the guests were in uniform, and the value of the Army red from a picturesque point of view was never more vividly demonstrated. The music was good, but, with all due respect to it, the company voted the talk even better, and the occasion was in all respects a notable one. Among representative people who were present were the Dowager Countess of Airlie; the Countess of Minto; Lady Ampthill, C.I.; Dame Ethel Becher, R.R.C.; Dame Maud McCarthy, R.R.C.; Dame Sydney Browne, R.R.C.; Lady Sloggett; the Hon. Lady Lawley; Lady Codrington; Mrs. Kerr Lawson; Miss Dowse, R.R.C.; Miss Taylor. There were more matrons of military hospitals than could be counted, and not a few from the civil hospitals.

---

The member for Bournemouth, General Croft, asked the Secretary of State for War last week whether he was aware that a great many trained nurses who served as nursing sisters on active service continuously from August 1914 had now been released from service without any official expression of gratitude for their services. The reply was, of course, that all nursing sisters on active service had received an official letter conveying thanks for their services on being demobilised. Everyone can appreciate the technical difficulties in the way of conveying the letters of thanks to some 20,000 nurses demobilised in many cases at lightning speed, who have often changed their addresses many times since and have failed to keep in touch with the Service. It would be next door to a miracle if all had received the thanks due to them, but we cannot believe many are really perturbed at the failure of the official expression of gratitude to reach them. Were it so it would be easy to bring the matter to the notice of the matron under whom they served, when the unintentional omission could be at once rectified.

---

The County Medical Officer of Health for Durham announces a dangerous shortage of district nurses for the needs of the million persons within his area. There are sixty health visitors, and should influenza appear as an epidemic he believes they could be utilised for help in organising relief measures of nursing, for providing for the care of delirious patients and for children whose mothers are ill. To assist in these measures Dr. Eustace Hill is trying to get together a body of home helps to work from 8 A.M. to 6 P.M. on six days in the week at a weekly wage of 20s. with food, or 32s. without. His success in this direction will be watched with great interest. The crux of the matter, however, is to train more nurses for the county and to guarantee them adequate salaries when they begin their career, so that they may have less temptation to drift away to other centres.

---

The Brentford Guardians have introduced a fifty-hour week in their infirmary, and have reached the prompt decision that a further addition to the Nurses' Home must be carried out without delay. The Ministry of Health has sanctioned the preliminary estimate of £30,000 for improving and extending the home, which will then be all that the nurses could desire. The Chairman comforted the economists on the board, who were inclined to think the nurses could wait for new quarters until more houses were built for the general public, with the information that the proposed outlay did not amount to more than a rate of an extra farthing in the pound.

## Figures and Tables

**Figure f1:**